# Direction-dependent interaction rules enrich pattern formation in an individual-based model of collective behavior

**DOI:** 10.1371/journal.pone.0198550

**Published:** 2018-06-14

**Authors:** Cole Zmurchok, Gerda de Vries

**Affiliations:** 1 Department of Mathematics, University of British Columbia, Vancouver, British Columbia, V6T 1Z2, Canada; 2 Department of Mathematical and Statistical Sciences, University of Alberta, Edmonton, Alberta, T6G 2G1, Canada; Texas A&M University System, UNITED STATES

## Abstract

Direction-dependent interaction rules are incorporated into a one-dimensional discrete-time stochastic individual-based model (IBM) of collective behavior to compare pattern formation with an existing partial differential equation (PDE) model. The IBM is formulated in terms of three social interaction forces: repulsion, alignment, and attraction, and includes information regarding conspecifics’ direction of travel. The IBM produces a variety of spatial patterns which qualitatively match patterns observed in a PDE model. The addition of direction-dependent interaction rules exemplifies how directional information transfer within a group of individuals can result in enriched pattern formation. Our individual-based modelling framework reveals the influence that direction-dependent interaction rules such as biological communication can have upon individual movement trajectories and how these trajectories combine to form group patterns.

## Introduction

Flocks of birds, schools of fish, and insect swarms are examples of collective behavior exhibited by animals. These groups of animals form spatial patterns as individuals coordinate their movements in efforts to search for a mate, forage for sustenance, or evade predation. It is scientifically important to understand the underlying decisions and rules that lead groups of individuals to form spatial patterns. For instance, it is often necessary to understand how to develop controls to these behaviors, in efforts to develop effective fishing strategies [1], to predict and prevent insect outbreaks [2], or to manage the movements of collections of robots [3].

In this paper, we focus on groups of individuals that are self-organized. We do not include external factors such as environmental conditions or spatial structures that lead to group formation, but instead focus on those spatial patterns that result from interactions between conspecifics. Within this context, there are two general methodologies for studying collective behavior. On the one hand, continuum models track the dynamics of the density of individuals throughout time and space. On the other hand, an individual-based approach can be taken, where a set of rules dictates the decision-making and subsequent movement of members of the population in question. A variety of analytical methods exist for continuum models, and the study of these types of models is quite active [4, 5]. Studies of individual-based models (IBMs) rely on computer simulations and qualitative results; however, a few quantitative methods, such as coarse-grained analysis [6, 7], are beginning to become prevalent.

Many IBMs of collective behavior are “three-zone models” which incorporate three social forces that govern the movements of individuals (repulsion, alignment, and attraction). These models seek to elucidate the mechanisms that may lead to collective behavior and pattern formation. For example, an IBM for schooling fish in three-dimensions in which individuals move in response to attraction, repulsion, and parallel orientation of nearby conspecifics can capture observed schooling behaviors [8, 9]. The simulated school forms spatial patterns such as a highly polarized group, a loosely polarized group, and captures the merging behavior of two smaller schools. Characteristics such as the degree of polarization (alignment of individuals within the school) and nearest neighbour distance match well with experimental data [9]. Some IBMs use decision-trees to govern the movement of the individuals [10]. A decision-tree describes how an individual’s response to conspecifics depends on a hierarchy of social interactions. In particular, using a stochastic decision-tree model [10] suggests that group cohesion is maximized by optimizing the size of a neutral zone between the repulsive and attractive social interaction forces. Individual-based models can also capture the transitions in group behavior related to minor changes in the individual interactions such as stochastic switching between states [6, 7], how the spatial position of individuals affects their influence on swarm behaviour [11], or how the history of the group structure influences the collective behavior as the interaction rules change [12].

Alongside modelling efforts, recent data-driven studies attempt to solve the inverse problem: given a set of movement trajectories of individuals, what are the interaction rules that led to these behaviors? To this end, it is possible to infer individual interaction rules from recorded trajectories of surf scoters, a species of waterfowl. A three-zone model with an additional frontal-zone interaction fits the surf scoter data best, and suggests that observed flocking behaviors can be explained by zonal models [13, 14]. The collective behavior of small schools of mosquitofish and golden shiners provide evidence for attractive-repulsion interactions mediated by speed, yet reveal a lack of evidence for explicit body-orientation (alignment) interactions [15, 16]. Visual information transfer best describes the underlying interaction network that connects signal emitters and receivers, while metric (interactions within a fixed distance) or topological (interactions with a fixed number of individuals) information can still explain the flow of information within a group [17]. In other species, such as starlings, three-dimensional reconstruction and simulations of collective flocking behaviour revealed that interactions do not depend on the metric distance between conspecifics, but rather topological distance—where a flocking individual interacts with a fixed number of individuals on average [18]. Nonetheless, these data-driven studies motivate the idea that interactions between individuals may be more subtle than simple repulsion, alignment, and attraction interactions, but may include specific frontal interaction zones [13], density-dependent speed interactions [15, 16], or visual information transfer [17]. Additionally, many examples of communication can be found in biological systems. The movement of Mormon crickets, for example, is influenced by the signals perceived from conspecifics moving in the same direction as the target individual [19]. In some species of fish, neighbours directly ahead are used to guide movements in place of fish directly adjacent [20], and some species of birds use directional sound signals to coordinate movements where the signal receiver must be faced by the signal emitter [21].

Direction-dependent animal communication mechanisms were proposed in [22–24] as an important ingredient in pattern formation. In this body of work, the authors superimpose mechanisms that describe not only how much information an individual receives, but also the manner in which that information is received onto the traditional repulsion-alignment-attraction social interaction zone framework [22]. Direction-dependent communication mechanisms are incorporated through a variety of submodels, which each consider a different mechanism of information transfer. As a result of these direction-dependent mechanisms, novel spatial patterns were discovered for models of this type, and the variety of group patterns could be explained within one modelling framework [22–24]. Moreover, in continuum three-zone PDE models, pattern formation can be studied through linear stability [5, 22, 23] and weakly nonlinear analysis [24]. An extension to the PDE model with density-dependent movement speeds was used to characterize the mechanisms that lead to the formation of very dense groups [24, 25]. A two-dimensional (2D) analogue to the PDE model is presented and studied in [26], and was later extended to incorporate predator-prey interactions [27]. Selective repulsion and attraction interactions, which depend on the relative velocity of the signal receiver and emitter, have also been studied in a 2D IBM [28]. In this way, individuals differentiate between approaching and retreating conspecifics through these proposed interactions, and consequently a variety of spatial patterns are found [28].

For the purposes of investigating the effect of incorporating direction-dependent interaction rules on the formation of spatial patterns, we focus on IBMs for three reasons. Firstly, the effect of direction-dependent communication mechanisms on collective behavior has been examined in the context of continuum models [4, 22–25], but not fully in the realm of IBMs—selective attraction and repulsion mechanisms have been considered by [28] and the influence of one direction-dependent alignment mechanism on collective behavior has been studied through “coarse” bifurcation diagrams derived from simulated data [7]. Secondly, we seek to describe an alternative individual-based model framework to explore ideas introduced by Eftimie and co-authors [4, 22–25]. This is the main purpose of this investigation—to determine whether an individual-based model can reproduce the patterns found in the PDE model with direction-dependent interaction rules. This new modelling framework may lend itself to better incorporate results from data-driven studies as discussed above. We can explore the effects of mediating attractive-repulsion interactions by speed, including alignment interactions [15, 16], or the influence of visual information transfer [17] on pattern formation. Without biological data, such as spatial trajectories of individuals during aggregation events, we do not aim to infer the mechanisms behind collective behavior but rather we seek to elucidate how directional-dependent interaction rules may lead to the formation of spatial patterns. Thirdly, an individual-based approach permits insight into the individual movement trajectories and how these trajectories combine to form a group pattern.

The investigation of direction-dependent interaction rules mechanisms in IBM of collective behavior began in [29, 30]. We describe the development of the IBM by imposing social interaction kernels on a stochastic individual-based model, and define 5 submodels which prescribe different direction-dependent interaction rules. Numerical simulations of the IBM demonstrate the formation of spatial patterns analogous to those found in the analogous PDE model. We also find that the effect of a small change in the repulsion interaction kernel is only a small qualitative change in pattern formation. A parameter sweep of the model reveals the dependence of pattern formation on the choice of social interactions, and illustrates the wide variety of patterns formed by the IBM. The parameter sweep also reveals that the same pattern may be formed with different combinations of direction-dependent interaction rules and the social interaction forces (repulsion, alignment, and attraction). Moreover, the parameter sweep suggests that certain patterns only form under certain direction-dependent interactions and interaction forces (for example, breathers are only found with attraction and repulsion interactions mediated by direction-dependent interactions as in submodel M4). Density-dependent movement speed in the IBM results in groups that split and merge. Determining whether the patterns formed in the PDE model can also be formed in the IBM is the main purpose of this investigation. Confirming the correspondence between the IBM and the PDE model suggests that our individual-based framework is suited to further study in higher dimensions and for adaptation to specific biological phenomena.

## Methods

In this section, we formulate the IBM with direction-dependent interaction rules. Specifically, in Individual-Based Approach, we formulate a one-dimensional IBM that describes a population of right- or left- moving individuals, and how these individuals change direction. Individuals change direction in response to distant individuals via the social interaction forces. The social interaction forces and the incorporation of direction-dependent interaction rules are discussed in Social Interaction Forces.

### Individual-based approach

**Fig 1:**
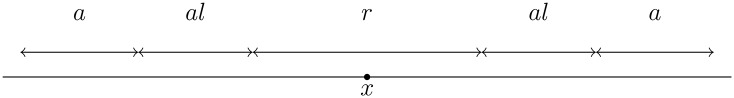
Social interaction zones. Cartoon depiction of the three social interaction zones surrounding an individual at location *x*. Repulsion (*r*) acts over short distances from the reference individual at *x*, alignment (*al*) over intermediate distances, and attraction (*a*) over longer distances. These zones may be disjoint, as illustrated, or may overlap.

Individual interactions with conspecifics are described through three social interaction forces: repulsion, alignment, and attraction. Repulsion describes the tendency for individuals at close distances to avoid each other, alignment describes the tendency for individuals to align with individuals at intermediate distances, and attraction describes the tendency for individuals to be attracted toward distant individuals. In one-dimension, the social interaction forces manifest as intervals surrounding a target individual at location *x*, as in Fig 1. Conspecifics in these intervals exert a social force and affect the movement of the target individual. For example, if many conspecifics are found in the zone of attraction of an individual, that individual will move towards the individuals in the zone of attraction. In contrast, if many individuals with the same velocity are found within the reference individual’s zone of alignment, that individual will tend to align their velocity of those individuals. The influence of incorporating alignment-based interactions versus repulsion- and attraction-based interactions is investigated in Spatial Pattern Formation.

An individual-based model, consisting of *N* individuals moving in one dimensional space, can be used to describe this social interaction mechanism. Following the development of the PDE model in [22–24, 31], but using a Lagrangian approach, we track the position and the direction of individuals moving on a line segment with periodic boundary conditions throughout time. Our approach is to develop a discrete-time stochastic individual-based model that has the same behaviour as the PDE model. Individual *i* has position *x*_*i*_(*t*) and direction *v*_*i*_(*t*) at time *t*, with $x_{i}\left(t\right)\in R$ and *v*_*i*_(*t*) = ±1. An individual with *v*_*i*_(*t*) = 1 (−1) is considered to be right- (left-)moving, and individuals change direction in response to conspecifics in their interaction zones. The social interaction forces felt by individual *i* are denoted by $y_{j,i}^{\pm }$, where the ± sign indicates whether an individual is right-moving (+) or left-moving (−), and *j* = *r*, *al*, *a* denotes repulsion (*r*), alignment (*al*), or attraction (*a*). A right- or left-moving individual *i* changes direction with rate $\lambda_{i}^{+}$ or $\lambda_{i}^{-}$, respectively, dependent on the social interaction forces:

$$\begin{array}{c} \lambda_{i}^{\pm }=\lambda_{1}+\lambda_{2}f\left(\alpha \left(y_{r,i}^{\pm }-y_{a,i}^{\pm }+y_{al,i}^{\pm }\right)\right), \end{array}$$ (1)

where the constants λ_1_ and λ_2_ describe a base-line turning rate and the bias turning rate, respectively, *α* is a scaling factor, and the turning function *f* is some dimensionless, bounded, and increasing function. Here, *f* is chosen to be a logistic function,

$$\begin{array}{c} f\left(x\right)=\frac{1}{1+e^{-2(x-y_{0})}}=\frac{1}{2}+\frac{\tanh(x-y_{0})}{2}, \end{array}$$ (2)

with *y*_0_ = 2. *y*_0_ is chosen so that in the absence of social interactions, *x* = 0, movement is dominated by random turning. With *y*_0_ = 2, *f*(0) is near zero, which implies $\lambda_{i}^{\pm }\approx \lambda_{1}$. Moreover, the repulsion and attraction social interaction forces enter the turning function with the opposite sign as these two interactions have opposite biological effects. Hence, the movement of individuals is influenced by both the base-line turning rate as well as the social interaction forces which factor into the bias turning rate. The scaling factor, *α*, ensures that we can translate between the different dimensions in the IBM (number of individuals) and the PDE model (density of individuals), so that the same parameter space can be used as in the PDE model (see Spatial Pattern Formation).

In each timestep, individuals first receive stimuli based on the social interaction forces and calculate their turning rate. They then update their position synchronously by moving in their direction of travel with constant speed *γ*. Lastly, individuals change direction if their probability of turning, $\lambda_{i}^{\pm }\Delta t$, is sufficiently large. This adds stochasticity to the IBM so that individuals may not always change direction. The algorithm for updating position and direction is as follows:

1. Calculate $\lambda_{i}^{\pm }$.
2. Change position and direction:
  $$\begin{array}{c} x_{i}\left(t+\Delta t\right)=x_{i}\left(t\right)+\gamma v_{i}\left(t\right)\Delta t, \end{array}$$ (3)
  $$\begin{array}{c} v_{i}\left(t+\Delta t\right)=\left\{ \begin{array}{cc} -v_{i}\left(t\right), & \text{if}\;\lambda_{i}^{\pm }\Delta t\geq X,\\ v_{i}\left(t\right), & \text{otherwise}, \end{array}\right. \end{array}$$ (4)
  where *X* is a uniformly distributed random variable on [0, 1], updated at each timestep. Individual positions and velocities are updated synchronously.

To complete the model development, the social interaction forces and direction-dependent interaction rules need to be described. How an individual measures the social interaction forces is described in the next section, Social Interaction Forces and Communication Mechanisms. Direction-Dependent interaction rules describe which signals and how much of those signals, are received by an individual.

### Social interaction forces and direction-dependent interaction rules

**Fig 2:**
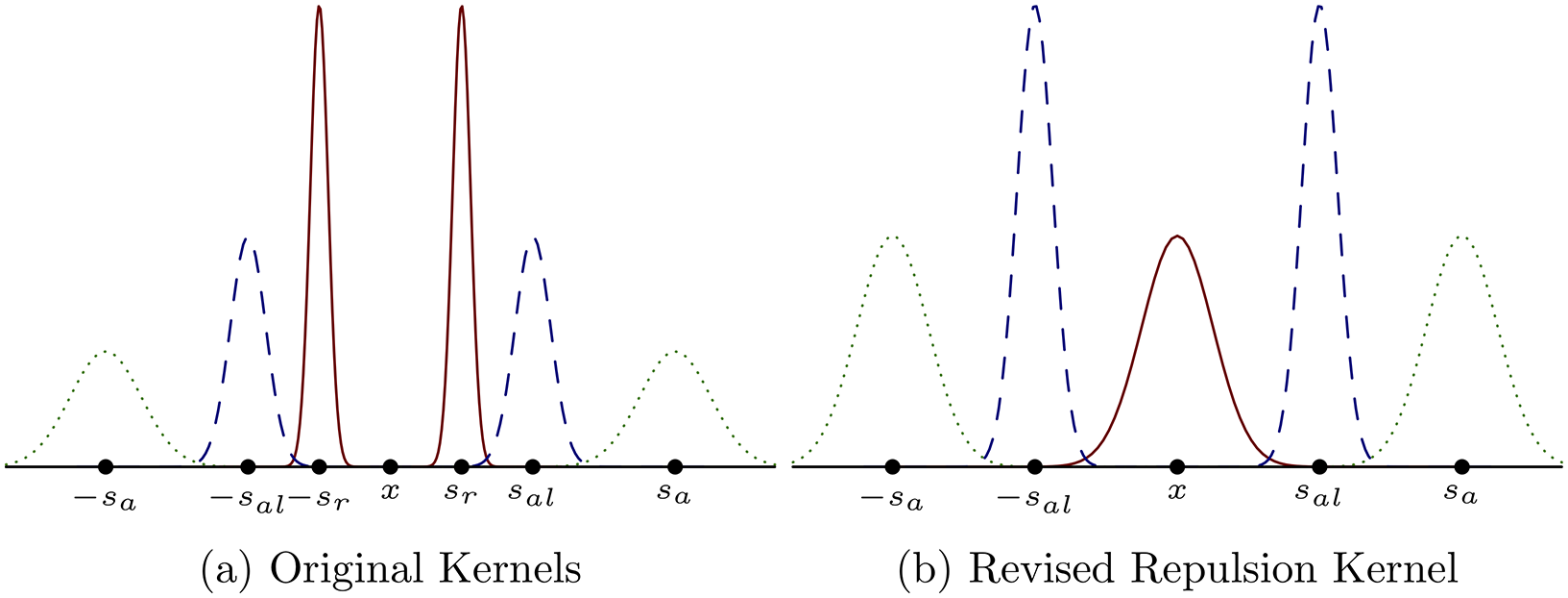
The social interaction kernels, *K*_*j*_(*s*), for *j* = *r*, *al*, *a*. In (a), the repulsion kernel (red, solid) weights conspecifics close to the target individual strongly, the alignment kernel acts for intermediate distances (blue, dashed), and the attraction kernel acts on large distances (green, dotted). In (b), the repulsion kernel (red, solid) is centered over the target individual, adding biological realism as the conspecifics very close to the target individual are weighted most heavily for the repulsion social interaction force. The alignment (blue, dashed) and attraction (green, dotted) kernels remain unchanged.

To calculate the social interaction forces, translated Gaussian kernels, *K*_*j*_(*x*), *j* = *r*, *al*, *a*, are chosen to weight the influence of individuals in each of the social interaction zones. The choice of these social interaction kernels is inspired by the choice of the interaction kernels in the corresponding PDE model [22–24]:

$$\begin{array}{c} K_{j}\left(s\right)=\frac{1}{\sqrt{2\pi m_{j}^{2}}}\exp\left(\frac{-{\left(s-s_{j}\right)}^{2}}{2m_{j}^{2}}\right), \end{array}$$ (5)

where *s*_*j*_ describes the centre of the Gaussian kernel and *m*_*j*_ describes the width (*j* = *r*, *al*, *a*). In practice, these kernels are truncated to avoid infinite ranges for the social interaction forces. The parameters *s*_*j*_ and *m*_*j*_ are chosen to organize the social interaction zones in a biologically meaningful way. Parameter values are summarized in Table 1; Gaussian kernels are illustrated in Fig 2(a). The choice of translated Gaussian kernels follows the development of the PDE model [22, 23], and is equivalent with classical choices for interaction kernels for attraction and repulsion [32]. Some models for collective behaviour permit more overlap between the social interaction zones [5]; however, in the IBM, we choose parameters for the alignment kernel so that it only has a small amount of overlap with the repulsion and attraction kernels.

**Table 1:** Social interaction kernel parameter values.

Kernel	*s*_*j*_	*m*_*j*_
Repulsion	0.25	0.03125
Alignment	0.5	0.0625
Attraction	1	0.125

Parameter values for the Gaussian social interaction kernels shown in Fig 2(a). *s*_*j*_ describes the centre of each Gaussian kernel and *m*_*j*_ describes the width. Here, *m*_*j*_ = *s*_*j*_/8 for *j* = *r*, *al*, *a*.

**Fig 3:**
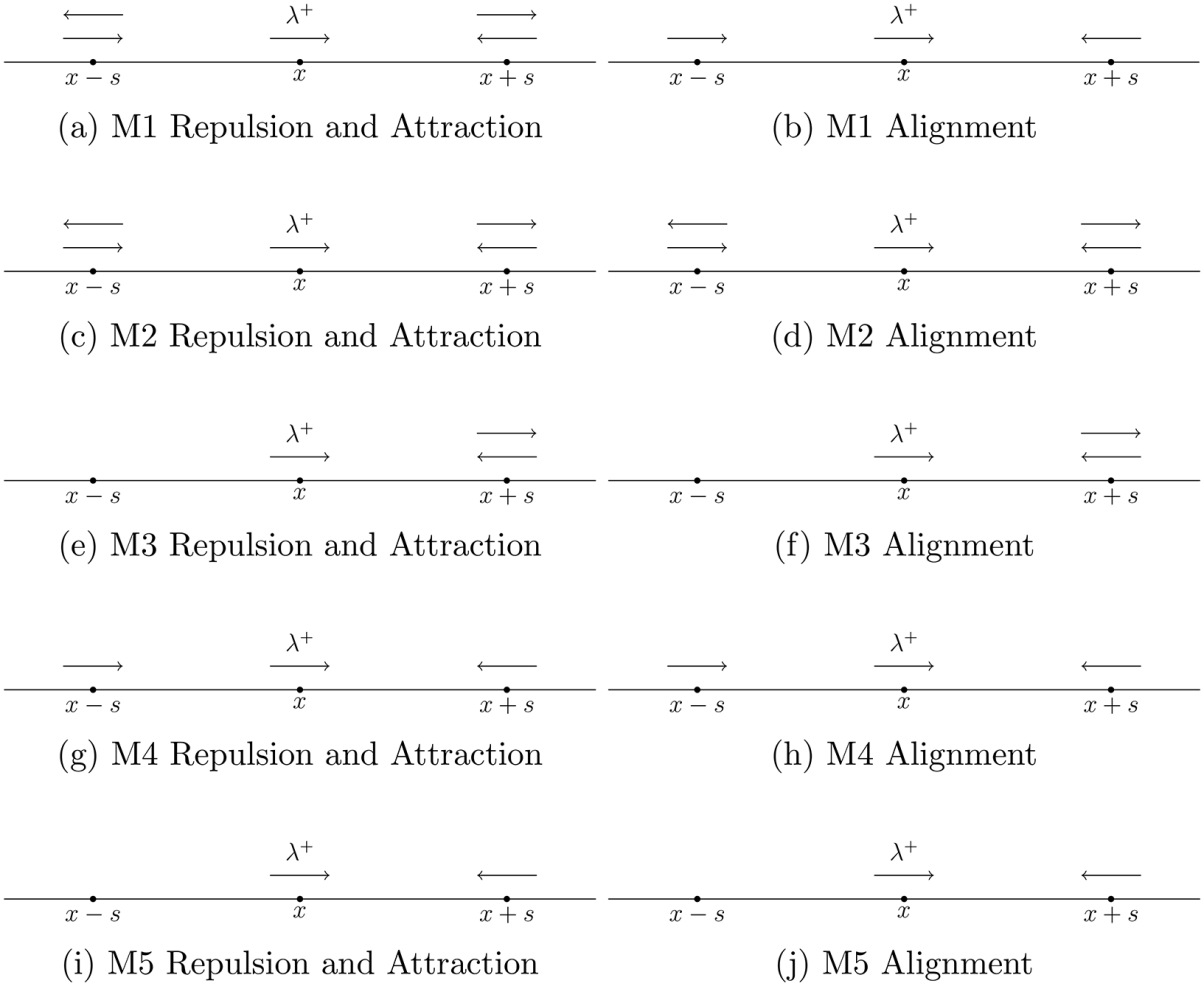
Cartoon depiction of the direction-dependent interaction rules in submodels M1 through M5. A right-moving reference individual at *x* receives signals from distant individuals, to the right at *x* + *s*, and from the left at *x* − *s*. Arrows at *x* + *s* and *x* − *s* indicate whether the reference individual will receive stimuli from distant right-moving (right arrow) and distant left-moving (left arrow) neighbours. For example, the direction-dependent interaction rules in submodel M1 describe how a right-moving reference individual at *x* receives signals from distant individuals, to the right at *x* + *s* and from the left at *x* − *s*. For attraction and repulsion (a), this individual uses all information from neighbours regardless of their direction of travel. Arrows pointing left and right indicate this. For alignment (b), the reference individual only uses information from neighbours heading toward it, as indicated by the arrows heading toward the reference individual. The submodels illustrated here are the same as those considered in [22–24].

The formula for $y_{j,i}^{\pm }$, *j* = *r*, *al*, *a*, not only depends on the weighting of conspecifics in the interaction zones as described above, but also on how information between individuals in interaction zones is shared. A direction-dependent interaction rule could, for example, be purely directional, and individuals could only receive information from individuals traveling towards them. That is, with this interaction rule, we are not only counting all individuals in a certain zone, but also taking direction of travel into account. In this way, to calculate the social interaction forces, a target individual needs to distinguish not only the location of conspecifics, but also their direction of travel. By way of example, one could define a direction-dependent interaction rule to be that the target individual uses information from all neighbours in the repulsion and attraction zones, but only information from individuals heading toward it in the alignment zone, as illustrated in Fig 3(a) and 3(b). A right-moving reference individual *i* at *x*_*i*_ is surrounded by individuals to the right (*x* + *s*) and individuals to the left (*x* − *s*). For repulsion and attraction, the target individual uses information from all neighbours in these zones as indicated by the right and left moving arrows in Fig 3(a). For alignment, the target individual only uses information from conspecifics moving toward it as indicated by right facing arrow at *x* − *s* and by the left facing arrow at *x* + *s* in Fig 3(b). In [22–24], this is called submodel M1, and we will adopt this naming scheme. The interaction rules specified in submodel M1 are direction-dependent as information transfer between individuals depends on the direction in which conspecifics are traveling. The social forces for submodel M1 thus need to reflect this direction-dependent interaction rule.

To incorporate the direction-dependent interaction rules as described in submodel M1 in the social forces, $y_{j,i}^{\pm }$, *j* = *r*, *al*, *a*, we consider how a reference individual *i* at *x*_*i*_ receives information from conspecifics in its social interaction zones. For repulsion and attraction, this individual uses information about all neighbours in its repulsion and attraction zones, regardless of the neighbours’ direction of travel. For alignment, this individual uses information about only those neighbours who are traveling toward it in the alignment zone. Define *q*_*j*_, *j* = *r*, *al*, *a*, to be the magnitude of the repulsive, alignment, and attractive force, respectively. Individual *i* at *x*_*i*_ experiences a *repulsive force*, $y_{r,i}^{\pm }$, given by

$$\begin{array}{c} y_{r,i}^{\pm }=\pm q_{r}\sum_{j\in Z_{r,i}^{R}}K_{r}\left(\right|x_{i}-x_{j}\left|\right)\mp q_{r}\sum_{j\in Z_{r,i}^{L}}K_{r}\left(\right|x_{i}-x_{j}\left|\right), \end{array}$$ (6)

where *K*_*r*_(*s*) is defined in (5), and $Z_{r,i}^{R,L}$ represents individual *i*’s zone of repulsion, to the right (superscript *R*) or to the left (superscript *L*). The + and − superscripts represent the direction of the target individual *i*, and the ± and ∓ signs are used to compare the social interaction forces between left (*L*) and right (*R*) zones. Similarly, as information is used from all neighbours in the zone of attraction, individual *i* at *x*_*i*_ then experiences an *attractive force*, $y_{a,i}^{\pm }$, given by

$$\begin{array}{c} y_{a,i}^{\pm }=\pm q_{a}\sum_{j\in Z_{a,i}^{R}}K_{a}\left(\right|x_{i}-x_{j}\left|\right)\mp q_{a}\sum_{j\in Z_{a,i}^{L}}K_{a}\left(\right|x_{i}-x_{j}\left|\right), \end{array}$$ (7)

where $Z_{a,i}^{R,L}$ represents individual *i*’s zone of attraction to the right (*R*) or to the left (*L*). The repulsive and alignment forces compare the strength of the forces ahead and behind the reference individual, as it is biologically relevant for an individual be repelled by nearby individuals or to be attracted to distant individuals, regardless of their direction of travel. On the other hand, the alignment forces compare the strength of forces exerted by right- or left-moving individuals (subject to the direction-dependent interaction rule), as the biological meaning of alignment is to adjust one’s direction to match that of neighbours at an intermediate distance. Since individual *i* uses information about only those neighbours who are traveling toward it in the alignment zone, the *alignment force* selects only those individuals who are left-moving (*v*_*j*_ < 0) to the right (*R*) of individual *i*, or those individuals who are right-moving (*v*_*j*_ > 0) to the left (*L*) of individual *i*. Thus, the alignment force is given by

$$\begin{array}{c} y_{al,i}^{\pm }=\pm q_{al}\sum_{\begin{array}{c} j\in Z_{al,i}^{R}\\ v_{j}<0 \end{array}}K_{al}\left(\right|x_{i}-x_{j}\left|\right)\mp q_{al}\sum_{\begin{array}{c} j\in Z_{al,i}^{L}\\ v_{j}>0 \end{array}}K_{al}\left(\right|x_{i}-x_{j}\left|\right), \end{array}$$ (8)

where $Z_{al,i}^{R,L}$ represents individual *i*’s zone of attraction to the right (*R*) or to the left (*L*).

Direction-dependent interaction rules are not restricted to the mechanism described as submodel M1. In [22–24] and [29, 30], submodel M1 is one of five direction-dependent interaction rules considered. These five submodels capture various information-receiving mechanisms, and exemplify how different environmental or biological constraints may be incorporated into this model. The submodels are defined as follows:

M1: the repulsion and attraction social interaction forces depend on information from all individuals in the repulsion and attraction zones, respectively; however, the alignment social interaction force only depends on individuals heading towards the reference individual (as above, see Fig 3(a) and 3(b));
M2: all three social interactions depend on all individuals, regardless of their direction of travel within the social interaction zones (see Fig 3(c) and 3(d));
M3: all three social interaction forces depend only on the information received from individuals ahead of the reference individual (see Fig 3(e) and 3(f));
M4: all three social interaction forces depend on the information from the zones ahead and behind, but only from those neighbours who are moving toward the reference individual (see Fig 3(g) and 3(h));
M5: all three social interaction forces depend only on the information from individuals ahead and moving toward the reference individual (see Fig 3(i) and 3(j)).

The social interaction forces for each submodel considered in this paper are described in Table 2.

**Table 2:** Submodels and social interaction forces.

Submodel	Repulsion & Attraction interaction force Alignment interaction force
M1	$y_{r,a,i}^{\pm }=\pm q_{r,a}\sum_{j\in Z_{r,a,i}^{R}}K_{r,a}\left(d_{ij}\right)\mp q_{r,a}\sum_{j\in Z_{r,a,i}^{L}}K_{r,a}\left(d_{ij}\right)$ $y_{al,i}^{\pm }=\pm q_{al}\sum_{\begin{array}{c} j\in Z_{al,i}^{R}\\ v_{j}<0 \end{array}}K_{al}\left(d_{ij}\right)\mp q_{al}\sum_{\begin{array}{c} j\in Z_{al,i}^{L}\\ v_{j}>0 \end{array}}K_{al}\left(d_{ij}\right)$
M2	$y_{r,a,i}^{\pm }=\pm q_{r,a}\sum_{j\in Z_{r,a,i}^{R}}K_{r,a}\left(d_{ij}\right)\mp q_{r,a}\sum_{j\in Z_{r,a,i}^{L}}K_{r,a}\left(d_{ij}\right)$ $y_{al,i}^{+}=q_{al}\left(\sum_{\begin{array}{c} j\in Z_{al,i}^{R}\\ v_{j}<0 \end{array}}K_{al}\left(d_{ij}\right)+\sum_{\begin{array}{c} j\in Z_{al,i}^{L}\\ v_{j}<0 \end{array}}K_{al}\left(d_{ij}\right)-\sum_{\begin{array}{c} j\in Z_{al,i}^{R}\\ v_{j}>0 \end{array}}K_{al}\left(d_{ij}\right)-\sum_{\begin{array}{c} j\in Z_{al,i}^{L}\\ v_{j}>0 \end{array}}K_{al}\left(d_{ij}\right)\right)$ $y_{al,i}^{-}=q_{al}\left(\sum_{\begin{array}{c} j\in Z_{al,i}^{L}\\ v_{j}>0 \end{array}}K_{al}\left(d_{ij}\right)+\sum_{\begin{array}{c} j\in Z_{al,i}^{R}\\ v_{j}>0 \end{array}}K_{al}\left(d_{ij}\right)-\sum_{\begin{array}{c} j\in Z_{al,i}^{L}\\ v_{j}<0 \end{array}}K_{al}\left(d_{ij}\right)-\sum_{\begin{array}{c} j\in Z_{al,i}^{R}\\ v_{j}<0 \end{array}}K_{al}\left(d_{ij}\right)\right)$
M3	$y_{r,a,i}^{+}=q_{r,a}\sum_{j\in Z_{r,a,i}^{R}}K_{r,a}\left(d_{ij}\right)$ $y_{r,a,i}^{-}=q_{r,a}\sum_{j\in Z_{r,a,i}^{L}}K_{r,a}\left(d_{ij}\right)$ $y_{al,i}^{+}=q_{al}\sum_{\begin{array}{c} j\in Z_{al,i}^{R}\\ v_{j}<0 \end{array}}K_{al}\left(d_{ij}\right)-q_{al}\sum_{\begin{array}{c} j\in Z_{al,i}^{R}\\ v_{j}>0 \end{array}}K_{al}\left(d_{ij}\right)$ $y_{al,i}^{-}=q_{al}\sum_{\begin{array}{c} j\in Z_{al,i}^{L}\\ v_{j}>0 \end{array}}K_{al}\left(d_{ij}\right)-q_{al}\sum_{\begin{array}{c} j\in Z_{al,i}^{L}\\ v_{j}<0 \end{array}}K_{al}\left(d_{ij}\right)$
M4	$y_{r,a,i}^{\pm }=\pm q_{r,a}\sum_{\begin{array}{c} j\in Z_{r,a,i}^{R}\\ v_{j}<0 \end{array}}K_{r,a}\left(d_{ij}\right)\mp q_{r,a}\sum_{\begin{array}{c} j\in Z_{r,a,i}^{L}\\ v_{j}>0 \end{array}}K_{r,a}\left(d_{ij}\right)$ $y_{al,i}^{\pm }=\pm q_{al}\sum_{\begin{array}{c} j\in Z_{al,i}^{R}\\ v_{j}<0 \end{array}}K_{al}\left(d_{ij}\right)\mp q_{al}\sum_{\begin{array}{c} j\in Z_{al,i}^{L}\\ v_{j}>0 \end{array}}K_{al}\left(d_{ij}\right)$
M5	$y_{r,a,i}^{+}=q_{r,a}\sum_{\begin{array}{c} j\in Z_{r,a,i}^{R}\\ v_{j}<0 \end{array}}K_{r,a}\left(d_{ij}\right)$ $y_{r,a,i}^{-}=q_{r,a}\sum_{\begin{array}{c} j\in Z_{r,a,i}^{L}\\ v_{j}>0 \end{array}}K_{r,a}\left(d_{ij}\right)$ $y_{al,i}^{+}=q_{al}\sum_{\begin{array}{c} j\in Z_{al,i}^{R}\\ v_{j}<0 \end{array}}K_{al}\left(d_{ij}\right)$ $y_{al,i}^{-}=q_{al}\sum_{\begin{array}{c} j\in Z_{al,i}^{L}\\ v_{j}>0 \end{array}}K_{al}\left(d_{ij}\right)$

The social interaction forces depend directly on the direction-dependent interaction rules prescribed by each submodel. Here, *d*_*ij*_ = |*x*_*i*_ − *x*_*j*_| is the distance between individuals *i* and *j*, *q*_*j*_, *j* = *r*, *al*, *a*, is the magnitude of the interaction force, *K*_*j*_, *j* = *r*, *al*, *a*, are the social interaction kernels, (5), and $Z_{j,i}^{R,L}$, *j* = *r*, *al*, *a*, describe individual *i*’s zone of repulsion, alignment, or attraction to the right (superscript *R*) or to the left (superscript *L*). The direction of individual *j*, *v*_*j*_, is necessary to distinguish between right- and left-moving individuals. The repulsive and alignment forces compare the number of neighbours ahead and behind the reference individual, as it is biologically relevant for an individual be repelled by nearby individuals or to be attracted to distant individuals. Alignment forces compare the number of right- or left-moving individuals (subject to the direction-dependent interaction rule), as the biological meaning of alignment is to adjust one’s direction to match that of neighbours at an intermediate distance.

This submodel paradigm allows for direction-dependent interaction rules to be varied beyond the five mechanisms proposed above and studied via simulations of the IBM. To incorporate direction-dependent interaction rules into the IBM, it is only necessary to specify which conspecifics a reference individual will interact with for the repulsive, alignment, and attractive social interaction forces.

### Varying interaction kernels

The choice of the social interaction kernels is biologically motivated, yet left to the modeller. Individuals should be repelled by conspecifics at close ranges to avoid collisions; individuals wish to orient their velocity with individuals at intermediate distances or distances that are “just right”; individuals wish to move toward distant individuals in order to remain in contact with them. The kernels discussed above do not adequately capture these ideas, as neighbours very close to an individual are not weighted as heavily in the repulsion interaction force as those neighbours who are located near ±*s*_*r*_. We modify the repulsion kernel to be centred about 0, as illustrated in Fig 2(b). Let ${\tilde{m}}_{r}=s_{r}/2$ and define

$$\begin{array}{c} {\tilde{K}}_{r}\left(s\right)=\frac{1}{\sqrt{2\pi {\tilde{m}}_{r}^{2}}}\exp\left(\frac{-s^{2}}{2{\tilde{m}}_{r}^{2}}\right). \end{array}$$ (9)

Using ${\tilde{K}}_{r}\left(s\right)$ as the repulsion kernel weights nearest neighbours more strongly than other individuals for the repulsion interaction force.

### Density-dependent speed

In this section, we modify the IBM to allow individuals to move with density-dependent speed. An assumption made earlier is that individuals move with constant speed *γ*. This may not be biologically realistic, as an individual’s movement speed would likely be influenced by conspecifics. For example, an individual may be motivated to slow down to avoid collision, or to speed up to join a group of distant conspecifics. Density-dependent speeds are considered in a two-dimensional IBM by [10] as well as work on extensions to the PDE model [4, 25–27, 31] and in other continuum models [5]. Moreover, [15] find that repulsion-based interactions are mostly mediated by changes in speed in schools of fish.

We adapt the IBM presented in Individual-Based Approach, and assume that individuals only change direction in response to alignment interaction forces. That is, a right- or left-moving individual *i* changes direction with rate $\lambda_{i}^{+}$ or $\lambda_{i}^{-}$, dependent only on the alignment interaction force, (8):

$$\begin{array}{c} \lambda_{i}^{\pm }=\lambda_{1}+\lambda_{2}f\left(y_{al,i}^{\pm }\right). \end{array}$$ (10)

Individuals adjust their speed by comparing individuals in the attraction and repulsion zones. It is biologically reasonable for social individuals to speed up to join distant groups, or to slow down to avoid collision with nearby individuals. We let individuals move with speed $\Gamma_{i}^{\pm }\left(y_{i}^{\pm }\right)$, where

$$\begin{array}{c} \Gamma_{i}^{\pm }\left(y_{i}^{\pm }\right)=\gamma \left(1+\tanh\left(y_{i}^{\pm }\right)\right), \end{array}$$ (11)

*γ* = 0.1 is a base-line movement speed, and $y_{i}^{\pm }=y_{a,i}^{\pm }-y_{r,i}^{\pm }$ compares the attraction and repulsion social interaction forces. To understand how the non-local speeds depend on individuals in the repulsion and attraction interaction zones, consider a right-moving individual *i* at *x*. For the sake of example, ignore conspecifics to the left of the individual. If $y_{a,i}^{+}>y_{r,i}^{+}$, then this individual senses a large number of individuals in its zone of attraction relative to the number of individuals in its zone of repulsion. Consequently, the individual will be motivated to speed up and join the distant group. This results in the speed of individual *i* to increase since $\Gamma_{i}^{+}\left(y_{i}^{+}\right)$ is an increasing function. Thus, the individual increases its speed to join the distant group of individuals. On the other hand, if $y_{a,i}^{+}<y_{r,i}^{+}$, the individual would be motivated to slow down to avoid collision. This is reflected in Eq (11). To incorporate this density-dependent speed into the IBM, we modify the position and velocity updating rule. In this case, in each time step, individuals make two adjustments: to their direction, and to their movement speed. The algorithm for updating position and velocity is as follows:

1. Calculate $\lambda_{i}^{\pm }$;
2. Change direction:
  $$\begin{array}{c} v_{i}\left(t+\Delta t\right)=\left\{ \begin{array}{cc} -v_{i}\left(t\right), & \text{if}\;\lambda_{i}^{\pm }\Delta t\geq X,\\ v_{i}\left(t\right), & \text{otherwise}; \end{array}\right. \end{array}$$ (12)
3. Calculate $\Gamma_{i}^{\pm }\left(y_{i}^{\pm }\right)$ using individual *i*’s new direction of travel, *v*_*i*_(*t* + Δ*t*);
4. Move with density-dependent speed:
  $$\begin{array}{c} x_{i}\left(t+\Delta t\right)=x_{i}\left(t\right)+\Gamma_{i}^{\pm }\left(y_{i}^{\pm }\right)v_{i}\left(t+\Delta t\right)\Delta t. \end{array}$$ (13)

The additional step of calculating the density-dependent movement speed using *v*_*i*_(*t* + Δ*t*) was not needed in the case of constant movement speed, as the individuals only respond to conspecifics by changing direction. In this case, the recalculation of social interaction forces is required to ensure that individuals do not erroneously respond to signals.

## Results

In this section, we simulate the model using a variety of direction-dependent interaction rules and a wide range of parameters. The previous investigation into the behavior of the PDE model revealed formation of spatial patterns [22–24]. Here, we aim to reproduce the spatial patterns previously seen in the analogous PDE model. This is the goal of Spatial Pattern Formation. In the remaining sections, Revised Repulsion Kernel, and Splitting and Merging Behvaior, we study the patterns produced by the IBM with the revised repulsion kernel and with density-dependent movement speed, respectively.

### Spatial pattern formation

*N* = 500 individuals are randomly scattered on a domain of length *L* = 10 with periodic boundary conditions. The initial positions of the individuals are selected from a uniform distribution on the domain. The social interaction kernels are truncated at 2*s*_*j*_, *j* = *r*, *al*, *a*, respectively, to avoid infinite interaction ranges. Individuals continue to move with a constant speed *γ* = 0.1. To ensure that we can translate between the parameter space in the analogous PDE model, we set the scaling factor $\alpha =\frac{AL}{N}$, where *A* = 2 is the total population size given in [22–24]. This normalization ensures that the magnitudes of the social interaction forces are scaled to match the choices of λ_1_, λ_2_, and the turning function, (2), in [22–24]. The normalization constant, in effect, scales the social interaction forces by the reciprocal of the density, $\frac{L}{N}$, then by *A* = 2, to match the total population size from [22–24].

**Fig 4:**
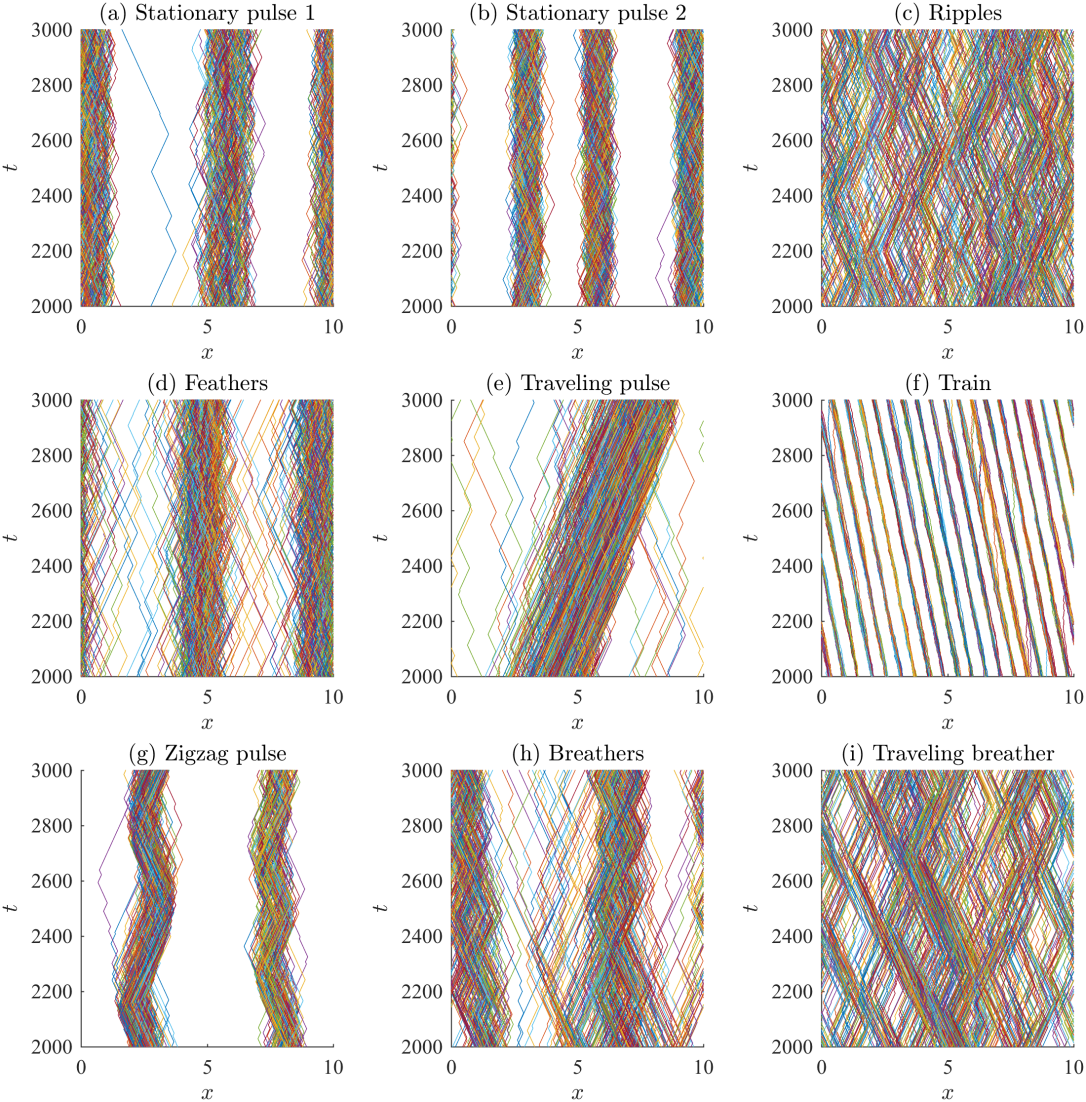
Examples of patterns obtained by various direction-dependent interaction rules. Parameters and rules (submodel) are described in Table 3, and boundary conditions are periodic.

**Fig 5:**
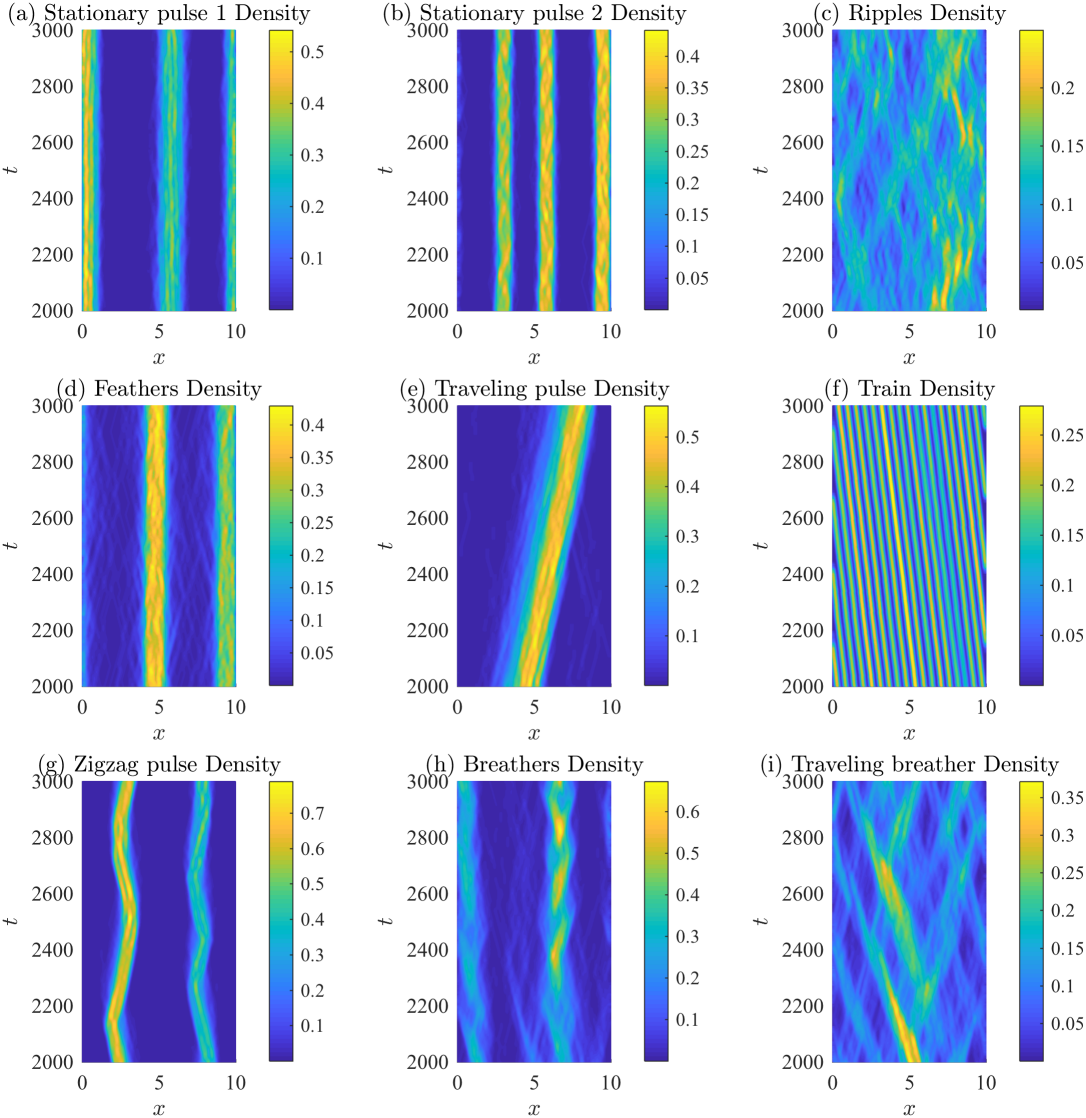
Density plots of patterns obtained by various direction-dependent interaction rules. Bright colours indicate high numbers of individuals, where the number density of individual is normalized by the total number of individuals. Density estimates are obtained via a kernel smoothing estimate from the corresponding trajectories in Fig 4. Subfigures correspond to the patterns shown in Fig 4. Parameters and rules (submodel) are described in Table 3.

Examples of the spatial patterns observed are shown in Fig 4. Individual trajectories are plotted using different colours, revealing the behavior of individuals throughout the domain. The initial transient dynamics have been eliminated, and the spatial patterns persist well beyond the time period presented. Initial conditions do not affect the qualitative patterns observed. Corresponding normalized density plots are shown in Fig 5. The density plots in Fig 5 allow the interior high- and low-density structure of the aggregations to be observed and for the patterns to be compared to those of the PDE model. A kernel smoothing method is used to generate the number density plots from the individual trajectories, and then the density is normalized by the total number of individuals (so the maximum density is unity). The kernel smoothing method estimates the density of individuals through space at time *t*, *ρ*(*x*, *t*), as

$$\begin{array}{c} \rho \left(x,t\right)=\frac{1}{Nh}\sum_{i=1}^{N}K\left(\frac{x-x_{i}\left(t\right)}{h}\right), \end{array}$$ (14)

where $K\left(x\right)=\exp(-x^{2}/2)/\sqrt{2\pi }$ is a standard normal distribution, *x*_*i*_(*t*) are the positions of each of the *n* individuals, and *h* is a smoothing parameter (fixed at *h* = 0.1).

Stationary pulses consist of an aggregation that does not travel and can have high-density subgroups, or have constant internal density. Figs 4(a) and 5(a) show stationary pulses with high-density subgroups, and Figs 4(b) and 5(b) show stationary pulses with constant internal density. Ripples are formed when left-moving and right-moving groups of individuals intersect but continue moving apart. Figs 4(c) and 5(c) show the formation of the ripple pattern. Figs 4(d) and 5(d) show stationary pulses that lose individuals from the edges of the groups, but eventually rejoin a group. This creates a feather-like pattern as individuals move away from the larger stationary pulse. A traveling pulse is a large group of individuals that travels together; whereas, a traveling train consists of multiple small groups of individuals that travel together. An example traveling pulse is shown in Figs 4(e) and 5(e). Figs 4(f) and 5(f) reveal multiple small groups moving across the domain in the traveling train pattern. When a group of individuals behaves like a traveling pulse but reverses direction sporadically, a zigzagging pulse is formed, as in Figs 4(g) and 5(g). Aggregations that expand and contract over time are called breathers. Breathing patterns can be stationary, as in Figs 4(h) and 5(h), or can travel across the domain, as in Figs 4(i) and 5(i). The spatial patterns formed by the IBM are not transient, but persist as quasi-stationary patterns. Simulations of the model for large time-steps reveal that the group patterns persist after some transient dynamics that occur as the spatial patterns form.

Simulations of the IBM with the parameter values in Table 3 provide specific examples of well-defined patterns that match the PDE model. To ensure the IBM matches the behavior of the PDE model throughout parameter space, numerical simulations of the IBM were performed using the same parameter spaces described in [31]:

**Case (a):** Repulsion and attraction only. In this case, the fixed parameters are *q*_*al*_ = 0, *γ* = 0.1, λ_1_ = 0.2, and λ_2_ = 0.9. The magnitude of the repulsive and attractive social interaction forces, *q*_*r*_ and *q*_*a*_, are varied, with (*q*_*r*_, *q*_*a*_) ∈ [0.5, 9].
**Case (b):** Alignment only. In this case, the fixed parameters are *q*_*r*_ = *q*_*a*_ = 0, *γ* = 0.1, and the influence of turning rates is investigated. Set $\lambda_{1}=\frac{0.2}{\alpha }$ and $\lambda_{2}=\frac{0.9}{\alpha }$. *α* and *q*_*al*_ are varied, with *α* ∈ [0.006, 1] and *q*_*al*_ ∈ [0.5, 10].
**Case (c):** All social interactions. In this case, we fix *γ* = 0.1, λ_1_ = 0.2, and λ_2_ = 0.9. The magnitudes of the social interaction forces are varied, with (*q*_*r*_, *q*_*al*_, *q*_*a*_) ∈ [1, 10].

**Table 3:** Parameter values produce spatial patterns.

Pattern	Submodel	λ_1_	λ_2_	*q*_*r*_	*q*_*al*_	*q*_*a*_
Stationary pulse 1	M1	0.2	0.9	2.4	0	2
Stationary pulse 2	M2	0.2	0.9	0.5	0	4
Ripples	M5	0.2	0.9	1.1	2	1.5
Feathers	M3	0.2	0.9	6.4	0	6
Traveling pulse	M1	0.2	0.9	0.5	2	1.6
Traveling train	M3	6.67	30	0	2	6
Zigzag pulse	M2	0.2	0.9	1	2	6
Breathers	M4	0.2	0.9	1	0	2
Traveling breathers	M4	0.2	0.9	4	2	4

Parameter values and submodels that produce spatial patterns. In this study, fixed model parameters are *N* = 500, *L* = 10, *A* = 2, Δ*t* = 0.05 and *γ* = 0.1.

Each of these parameter regimes was investigated using the direction-dependent interaction rules described by submodels M1 through M5. Numerical simulations for the described range of parameters were completed for each submodel, M1 through M5, for each parameter case. To determine if the parameter case would form specific spatial patterns, the results from the simulations were qualitatively compared to patterns in Figs 4 and 5. The results of this investigation are described in Table 4 by indicating which patterns form as a result of considering a certain submodel and parameter regime (Case (a), (b), or (c)). For example, the interaction rules described by submodel M1 with parameters in case (a) can produce stationary pulses. Submodel M1 with parameters in case (b) can produce 2 different types of behavior, namely stationary pulses, traveling trains. Submodel M1 with parameters in case (c) can produce stationary pulses, traveling pulses, and zigzag pulses. Dashes indicated that the particular pattern has not been observed for that range of parameters, but does not rule out the formation of a specific type of pattern for a specific submodel. This investigation suggests the importance of alignment in the formation of moving groups. Traveling groups, such as the traveling pulse and traveling train, appear to require non-zero alignment forces. Traveling breathers can be produced without alignment; however, in this case, attraction and repulsion have the same magnitude. This allows individuals to escape the group and subsequently rejoin, leading to the expansion and contraction of the group.

**Table 4:** Patterns produced by the IBM.

Model	Stat. Pulse	Ripples	Feathers	Trav. pulse	Trav. train	Zigzag	Breathers	Trav. breathers
M1	(a),(b),(c)	-	-	(c)	(b)	(c)	-	-
M2	(a),(c)	-	-	(c)	(c)	(c)	-	-
M3	-	-	(a),(c)	(b),(c)	(b)	-	-	-
M4	(b)	-	(c)	-	(b)	-	(a)	(a)
M5	(b)	(a),(c)	-	-	-	-	-	-

Patterns produced by the IBM for submodels M1 through M5. (a), (b) and (c) indicate that the corresponding pattern was produced by parameters from the corresponding parameter regime (see text). Dashes indicate that the parameter was not observed for any of the parameter regimes.

The parameter investigation reveals that the PDE model and the IBM model generally have the same behavior. Pattern formation of the PDE model is summarized in Table 5.3 in [31] across parameter space and the five submodels. Comparing the patterns formed by the IBM (Table 4) with those patterns formed by the PDE model demonstrates that the two modelling approaches reveal the same group formation patterns.

### Revised repulsion kernel

**Fig 6:**
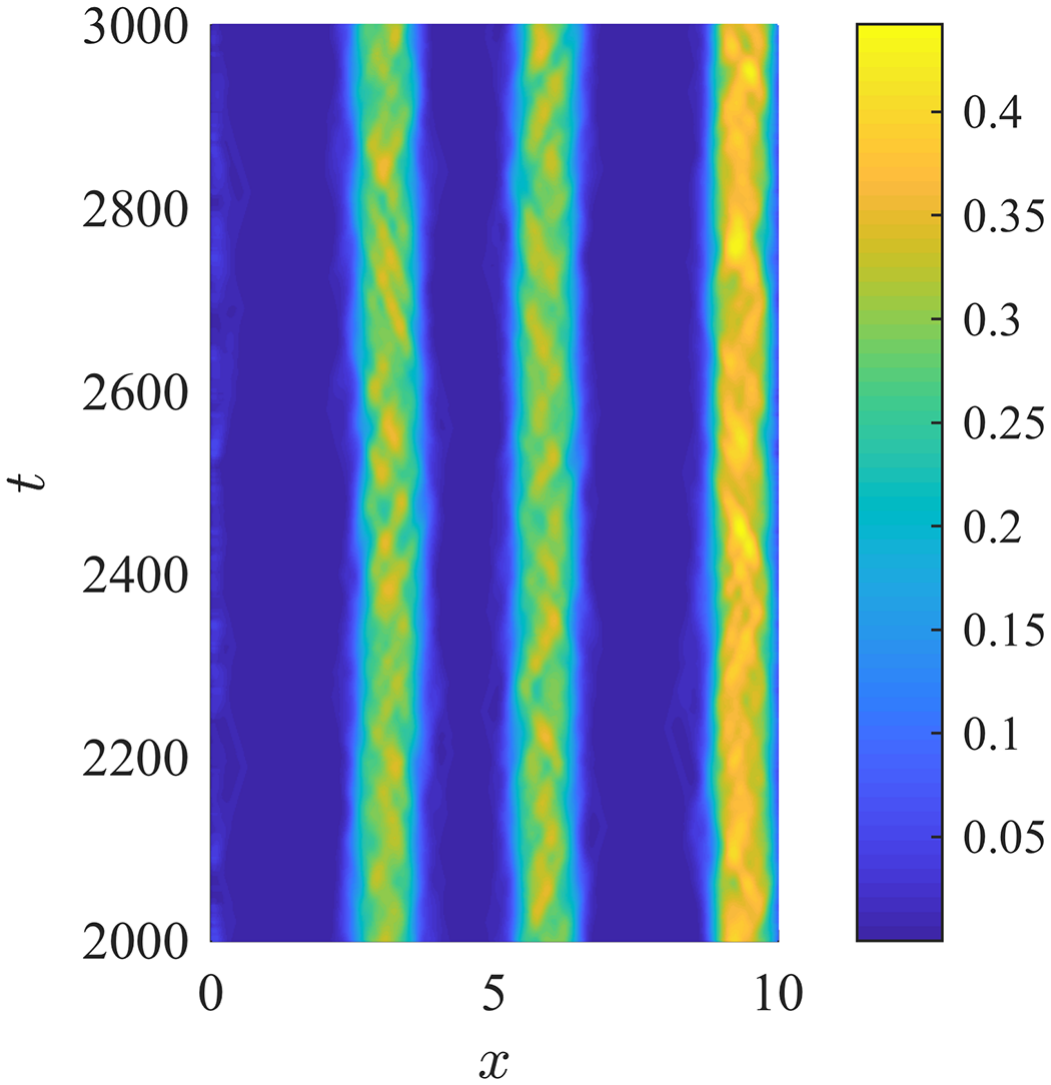
Density plot of a stationary pulse formed using the revised repulsion kernel, ${\tilde{K}}_{r}\left(s\right)$. Bright colours indicate high numbers of individuals. The number density has been normalized to 1. Parameters are identical to those for the stationary pulse observed in Fig 5(a) (M1, λ_1_ = 0.2, λ_2_ = 0.9, *q*_*r*_ = 2.4, *q*_*al*_ = 0, *q*_*a*_ = 2). Note the loss of high-density subgroups within each stationary pulse. The revised repulsion kernel does not permit conspecifics to remain together as a small group without exerting a large repulsive force on each other.

Repulsion is more strongly weighted at closer distances to the target individual with the revised repulsion kernel ${\tilde{K}}_{r}\left(s\right)$. With the revised repulsion kernel, pattern formation is largely unaffected as simulations show the formation of the same patterns using the same parameters as in previous simulations. However, minor differences in pattern formation exist, such as with the stationary pulse formation. In simulations with the original repulsion kernel, small high density subgroups of individuals form within the pulse; however, these small high density groups of individuals do not form using the revised repulsion kernel (compare Fig 5(a) and 6). This results from the removal of the valley between the peaks formed by the original repulsion kernel (Fig 2), as previously, individuals sufficiently close could remain together as a group without exerting a large repulsive force on each other by aggregating in this valley. The revised repulsion kernel, ${\tilde{K}}_{r}\left(s\right)$, does not permit individuals to cluster this closely without exerting a strong repulsive force upon each other.

### Splitting and merging behavior

**Fig 7:**
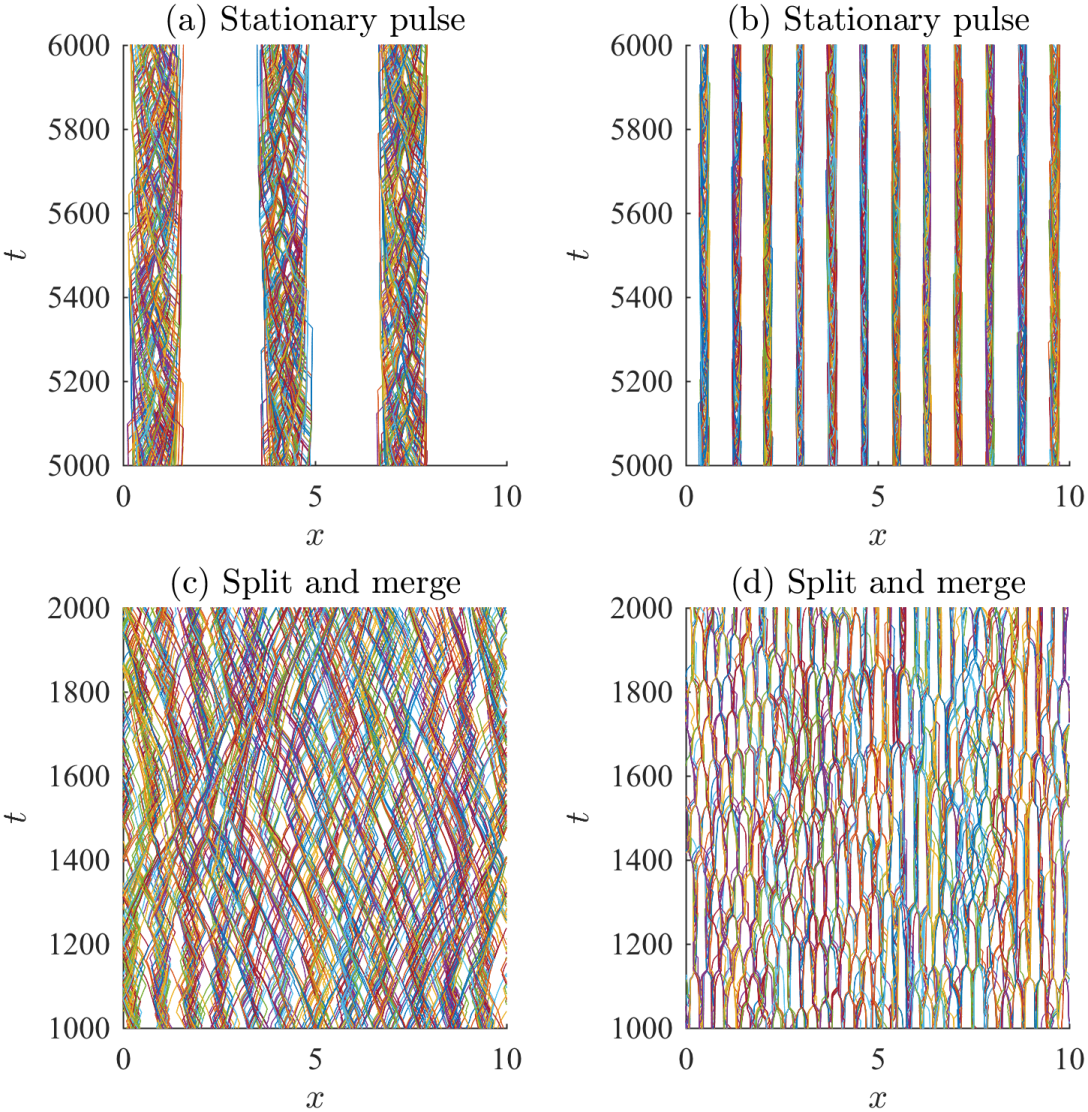
Splitting and merging behavior in the IBM with density-dependent speed results with or without alignment. Here, submodel M1 is used with *N* = 500, *L* = 10, Δ*t* = 0.05 and the boundary conditions are periodic. Other parameters are given in Table 5. Corresponding density plots in are shown in Fig 8. In (a) and (b), stationary pulses form. In (c) and (d), splitting and merging behavior is observed with (c) or without alignment (d).

We simulated the IBM with density-dependent speed to understand the behavior of the model, and observed stationary aggregations and groups that split and merge, similar to results found by [31] for the PDE model with density-dependent speed. We did not observe splitting and merging behavior in either the PDE model or IBM with constant movement speed in the parameter regimes explored, and occurs with or without alignment. Fig 7(a) and 7(b) show a high-density aggregation splitting and merging formed with *q*_*al*_ = 3.5. Fig 7(c) and 7(d) reveal small groups that split apart and merge with other small groups repeatedly, with *q*_*al*_ = 0.

**Table 5:** Parameter values with density-dependent speed.

Pattern	Submodel	λ_1_	λ_2_	*q*_*r*_	*q*_*al*_	*q*_*a*_
(a) Stationary Pulses	M1	0.2	0.9	0.1	0	0.5
(b) Stationary Pulses	M1	0.2	0.9	0.1	2	0.7
(c) Splitting and Merging	M1	0.2	0.9	0.1	3.5	0.2
(d) Splitting and Merging	M1	0.2	0.9	0.5	0	0.1

**Fig 8:**
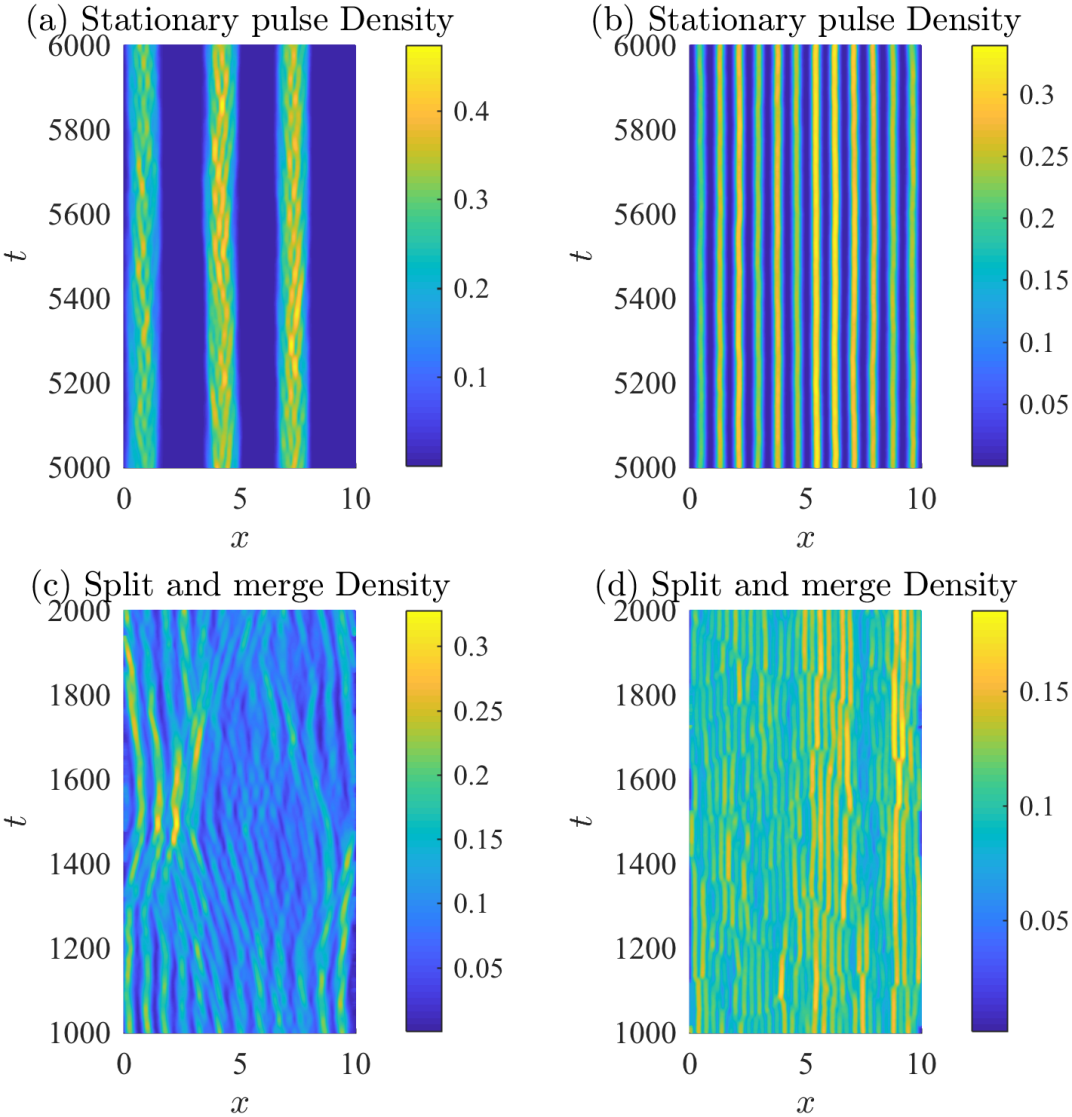
Density plots of patterns produced by the IBM with density-dependent speed. Bright colours indicate high numbers of individuals. The number density has been normalized to 1. Corresponding trajectories are shown in Fig 7.

Parameter values for patterns produced by the IBM model with density-dependent speed for the simulations shown in Figs 7 and 8.

## Discussion

Following the use of direction-dependent communication mechanisms as in [22–24], we formulated the IBM on a one-dimensional domain with periodic boundary conditions, upon which individuals can move to the right or left. Direction-dependent interaction rules were superimposed upon the traditional social interactions of repulsion, alignment, and attraction. These direction-dependent interaction rules describe how and how much information is received by a target individual from conspecifics. A variety of interaction rules can be imagined, but only five are considered as submodels. Our individual-based approach describes how the ideas introduced by [4, 22–25] influence the movement of individuals as their trajectories combine to produce group patterns.

The spatial patterns form as a result of various submodels and varied parameter space. The IBM reproduces classical patterns, such as stationary aggregations, ripples, traveling pulses, and traveling trains, and produces novel patterns such as feathers, zigzag pulses, breathers, and traveling breathers. The spatial patterns observed here are noisier than the analogous patterns formed in the PDE model from [22–24]. In the IBM, individuals change direction if their turning probability, $\lambda_{i}^{\pm }\Delta t$, is larger than a uniformly distributed random variable. The effect of stochasticity can be seen very well in the traveling train pattern (Fig 4(f)), as individuals will occasionally leave the group they have been traveling with and are subsequently absorbed by another group. In the zigzag pattern (Fig 4(g)), the majority of individuals travel with the main zigzagging group; however, a small number of individuals continue to travel past the turning main group. Eventually, they return to the main group after being attracted by the large number of distant individuals. We did not determine the influence of demographic stochasticity (i.e., group size or group heterogeneity) on the formation of patterns, explore the impact of adjusting other model parameters such as Δ*t* on the results, nor other boundary conditions. We hypothesize that there is a critical group size below which well-defined patterns may not form, as social interaction forces may not reach sufficiently high magnitude to affect an individual’s turning rate. Increasing Δ*t* will increase the turning probability, $\lambda_{i}^{\pm }\Delta t$, and likely create patterns with less noise.

The choice of social interaction kernels, although biologically motivated, is left to the modeller. The revised repulsion interaction kernel considered in Varying Interaction Kernels weights nearby individuals more strongly than the original social interaction kernels. Simulations with the revised repulsion kernel produce spatial patterns under matching parameter regimes as before and as in [22–24]. Small differences in pattern formation exist in simulations with the revised kernels. For example, small, high-density subgroups do not form within the stationary pulses with the revised repulsion kernels due to high repulsive forces at very short distances (see Fig 6).

Data-driven studies have elucidated the interaction rules and social forces which govern the decision-making of individuals in collective behavior [13, 15–17]. One of these studies finds that visual sensing most accurately predicts information transfer during leadership events in schools of fish, as compared to “standard” metric and topological interactions [17]. Here, our parameter investigation reveals that direction-dependent interactions which model visual information transfer (information from only ahead of the individual as in submodels M3 and M5), can produce a wide variety of patterns under a variety of parameter regimes as shown in Table 4. In flocks of surf scoters, an explicit frontal-zone of interaction with attractive-repulsion interactions that balances flock-wide interactions is necessary to best explain observed patterns [13]. This directional-preference to the single nearest neighbour directly ahead of the target individual also corroborates the inclusion of direction-dependent mechanisms.

The interaction rules of schooling fish have also been inferred by studying movement trajectories [15, 16]. In particular, only weak evidence for fish alignment is found. Here, our results agree as many spatial patterns can be found using only attraction and repulsion interactions (parameter case (a) in Table 4). Our results suggest that alignment interactions may be important in the formation of some patterns, such as travelling pulses, travelling trains, zigzag pulses. Moreover, splitting and merging behavior, which emerges due to the inclusion of density-dependent speed, can occur with or without alignment interactions. Repulsion-based interactions are mediated primarily by changes in speed instead of a directional change [15]. This highly motivates the study of density-dependent speed. Density-dependent interactions can lead to stationary aggregations and groups that split and merge as shown in Fig 7. In addition, we find that density-dependent speed based on attractive and repulsive interactions can lead to splitting and merging patterns, with and without direction changes due to alignment (Fig 7).

A multitude of spatial patterns can be observed within one modelling framework by exploring direction-dependent interaction rules. In particular, we are able to reproduce the spatial patterns observed in a PDE model with direction-dependent social interactions using an IBM framework. Nonetheless, our work has some caveats. First, while we focused solely on direction-dependent interaction rules in this investigation, it is possible that there are other means of information transfer among individuals in a group. Data-driven approaches to elucidate information transfer within groups should guide future modelling efforts [13, 15–17]. Second, natural biological aggregations rarely occur in one-dimension, and extending the IBM to two or three dimensions could allow for the model predictions to be compared with empirical data from aggregations in nature (as discussed briefly below). Third, the qualitative investigation presented here could be improved with the addition of quantitative data analysis. For example, the consideration of order parameters to quantify pattern formation, such as average nearest neighbour distance, group polarity [14], distance travelled, or the use of topological data analysis [33] could allow us to quantify the comparison of the IBM and PDE models and to examine the influence of stochasticity and demographic stochasticity on pattern formation.

We suggest that extending the IBM with direction-dependent interaction rules to two or three dimensions remains as a next step since these models better capture the physical reality of aggregations in nature. Romanczuk and Schimansky-Geier [28] studied a two-dimensional individual-based model where each individual can distinguish between conspecifics moving towards or away. They found that collective behavior arises without explicit velocity-alignment interaction, and derived an analytical condition to describe the onset of collective motion. To distinguish between individuals approaching and moving away from a target individual, the notion of of “relative velocity” which is calculated from the positions and velocities of interacted individuals was introduced. Using this notion of “relative velocity” and the framework described in [28], it would be possible to modify the social interaction forces to include direction-dependent interaction rules as considered in submodels M1-M5 here. Another possible extension of the direction-dependent IBM to two dimensions is to restrict the movement of individuals to a lattice, and “copy” the *x*-direction interactions from the IBM herein to the *y*-direction. With a modification to allow individuals to change from left- or right-moving to also change from up- or down-moving and vice versa, it would be possible to extend the direction-dependent interaction rules to a two-dimensional model where interactions are restricted to the four cardinal directions (up, down, left, right). To overcome this restriction, the social forces would need to be defined in circles or annuli surrounding a target individual (instead of intervals), and the direction-dependent interaction rules would again need a notion of “relative velocity” to determine which conspecifics are moving towards or away from a target individual. Indeed, Fetecau [26] has extended the 1D PDE model from [22–24] to two dimensions by adopting a kinetic formulation of a velocity-jump process for the dispersal of organisms. Fetecau describes an integro-differential equation for the total density of individuals in space moving in a certain direction, with the social interaction forces described through the reorientation rate and kernel. As our 1D IBM results are similar to the corresponding 1D PDE model, we expect that a 2D IBM which incorporates the same elements of the integro-differential equation model in [26] will produce similar collective behaviors.

In closing, the individual-based approach discussed in this paper reinforces the idea that direction-dependent interaction rules may play a significant role in pattern formation. The IBM recapitulates many of the ideas explored by Eftimie and co-authors [4, 22–25], and reveals how many individual trajectories can combine to form group patterns.

## Supporting information

S1 File A MATLAB implementation of the individual-based model is available as supplementary information. (M)
